# First-line CDK4/6 inhibitor treatment of HR-positive, HER2-negative metastatic breast cancer: real-world data from Ho Chi Minh City Oncology Hospital, Vietnam

**DOI:** 10.3389/fonc.2026.1871703

**Published:** 2026-09-07

**Authors:** Thi Hong Duc Phan, Hoang Quy Nguyen

**Affiliations:** 1 Department of Oncology, Pham Ngoc Thach University of Medicine, Ho Chi Minh City, Vietnam; 2 Department of Oncology, School of Medicine, University of Medicine and Pharmacy at Ho Chi Minh City, Ho Chi Minh City, Vietnam; 3 Department of General Internal Medicine, Ho Chi Minh City Oncology Hospital, Ho Chi Minh City, Vietnam

**Keywords:** CDK4/6 inhibitors, HER2-negative breast cancer, hormone receptor-positive breast cancer, metastatic breast cancer, overall survival, progression-free survival

## Abstract

**Objective:**

This study aimed to characterize the tumor response rate, progression-free survival (PFS), and overall survival (OS) among patients with HR-positive, HER2-negative metastatic breast cancer receiving CDK4/6 inhibitors plus endocrine therapy at Ho Chi Minh City Oncology Hospital. Secondary objectives included identifying clinical factors associated with survival outcomes and characterizing treatment-related toxicities.

**Materials and methods:**

We conducted a retrospective cohort study enrolling 107 patients who received first-line ribociclib or palbociclib in combination with an aromatase inhibitor or fulvestrant at Ho Chi Minh City Oncology Hospital between March 1, 2021, and April 1, 2023.

**Results:**

The median follow-up duration was 29 months. The median PFS was 28.0 months. The Kaplan–Meier-estimated PFS rates at 12 and 24 months were 66.4% (95% CI, 56.6%–74.4%) and 54.1% (95% CI, 44.2%–63.0%), respectively. The median OS was not reached; corresponding OS rates at 12 and 24 months were 88.8% and 87.8%, respectively. On multivariate Cox regression analysis, ER and PR expression levels emerged as independent prognostic determinants of PFS. Grade 3–4 neutropenia was observed in 58.8% of patients; all events were managed through dose interruption or reduction, and no cases of febrile neutropenia were documented.

**Conclusion:**

CDK4/6 inhibitors (ribociclib or palbociclib) combined with endocrine therapy demonstrated favorable efficacy and tolerability in Vietnamese patients with HR-positive, HER2-negative metastatic breast cancer. Treatment outcomes and safety profiles were consistent with those reported in pivotal clinical trials and real-world studies. These findings should be interpreted in light of the retrospective single-center design, modest sample size, non-randomized treatment allocation, and immature OS data.

## Introduction

Breast cancer ranks as the most prevalent malignancy among women globally, representing approximately 23.8% of all incident cancer cases and accounting for 15.4% of cancer-related deaths (GLOBOCAN, 2022) (1). The hormone receptor–positive, HER2-negative subtype, defined by ER and/or PR positivity in the absence of HER2 amplification, constitutes the most common breast cancer subtype, affecting approximately 60–70% of all patients (2). Endocrine therapy remains the mainstay of treatment for this subtype. Although antiestrogen therapy is often effective initially, approximately 50% of patients with hormone receptor-positive breast cancer eventually develop endocrine resistance (3, 4). Among patients with HR-positive, HER2-negative metastatic breast cancer, initial endocrine therapy confers superior PFS and OS compared with upfront chemotherapy, with hazard ratios of 0.83 (95% CI: 0.69–0.99) and 0.71 (95% CI: 0.58–0.86), respectively. CDK4/6 inhibitors (palbociclib, ribociclib, abemaciclib) have significantly improved outcomes in patients with advanced, metastatic hormone receptor-positive breast cancer compared with endocrine monotherapy (such as tamoxifen, fulvestrant, or aromatase inhibitor) by affecting the cell cycle. The PALOMA-2 study showed that palbociclib, combined with letrozole, prolonged median PFS to 24.8 months compared to 14.5 months in the endocrine-only group (5). Concordantly, MONALEESA-2 reported a median PFS of 25.3 months with ribociclib plus endocrine therapy, compared with 16.0 months in the placebo arm (6). MONARCH-3 similarly showed that abemaciclib produced a median PFS of 28.18 months versus 14.76 months for the control group (7). On the strength of these landmark trials, major international guidelines now recommend CDK4/6 inhibitor–based combination therapy as the preferred first-line treatment for HR-positive, HER2-negative advanced breast cancer in the absence of visceral crisis.

The clinical benefit of CDK4/6 inhibition in HR-positive, HER2-negative breast cancer has been further corroborated by large-scale real-world studies, including CompLEEment-1 and P-VERIFY. In Vietnam, CDK4/6 inhibitors became available beginning in 2021 (palbociclib: March 2021; ribociclib: May 2021) and are approved for use in combination with endocrine therapy for patients with hormone receptor–positive, HER2-negative locally advanced or metastatic breast cancer, thereby substantially expanding the therapeutic armamentarium for this disease. Although there have been many international studies on the efficacy and safety of CDK4/6 inhibitors combined with endocrine therapy in the first-line treatment of metastatic breast cancer, in Vietnam, there has been no internationally published study on first-line treatment with CDK4/6 inhibitors. Earlier single-center experiences with limited sample sizes were presented only at domestic conferences. At the SABCS 2023 conference, we first presented a poster reporting real-world outcomes of first-line treatment with CDK4/6 inhibitors in Vietnam (8). With extended follow-up, the present study aimed to evaluate the effectiveness and safety of first-line CDK4/6 inhibitors combined with endocrine therapy in Vietnamese patients with HR-positive/HER2-negative metastatic breast cancer at Ho Chi Minh City Oncology Hospital. We assessed PFS, OS, factors potentially associated with survival outcomes, and treatment-related toxicities. The question posed in our study is to understand the treatment outcomes of CDK4/6 inhibitors in the first-line treatment of HR+HER2- metastatic breast cancer at Ho Chi Minh City Oncology Hospital. To answer the above question, we conducted a study on the topic: “Evaluation of the efficacy and safety of CDK4/6 inhibitors combined with endocrine therapy in the first-line treatment of HR+HER2-negative metastatic breast cancer” to evaluate the results and draw treatment experiences with the objectives of determining PFS and OS of patients with HR+HER2-negative metastatic breast carcinoma treated with CDK4/6 inhibitors combined with endocrine therapy in the first-line treatment. Additionally, we examined several factors that may impact PFS and OS. Finally, we evaluated some toxicities of first-line treatment with CDK4/6 inhibitors combined with endocrine therapy in Vietnamese patients with HR+HER2-metastatic breast carcinoma.

## Materials and methods

### Patients

The retrospective study was done by reviewing and collecting data from the hospital database of patients at Ho Chi Minh City Oncology Hospital, Vietnam, from March 2021 to April 2023. Women with HR-positive, HER2-negative recurrent or metastatic breast cancer who had not received previous systemic therapy for advanced disease were eligible. In this study, first-line therapy referred to the first systemic treatment for recurrent or *de novo* metastatic breast cancer. All patients were CDK4/6 inhibitor–naïve and received a CDK4/6 inhibitor in combination with endocrine therapy as their first systemic regimen for advanced disease. Prior adjuvant endocrine therapy and/or chemotherapy in the early-stage setting were allowed and were systematically recorded. Premenopausal patients received ovarian function suppression according to institutional practice. Endocrine resistance was defined according to the 5th International Consensus Conference for Advanced Breast Cancer (ABC5) criteria as relapse during the first 2 years of adjuvant endocrine therapy, relapse within 12 months after completing adjuvant endocrine therapy, or disease progression within 6 months of first-line endocrine therapy for advanced disease. Patients who did not meet these criteria were classified as endocrine sensitive.

### Treatment

Palbociclib was administered at a dose of 125 mg/day for 3 weeks, followed by a 1-week drug holiday. Ribociclib was administered at a dose of 600 mg/day for 3 weeks, followed by a 1-week drug holiday. Letrozole was administered at a dose of 2.5 mg once daily. One 500 mg dose of fulvestrant was administered by intramuscular injection on days 1 and 15 of the first cycle and then on day 1 of each subsequent cycle (every 28 days). The dose was reduced based on the occurrence of adverse events. Among patients receiving ribociclib, electrocardiographic monitoring and liver function testing were performed at regular intervals consistent with institutional protocol.

### Safety monitoring

Patients receiving ribociclib underwent baseline electrocardiography (ECG), complete blood count, serum biochemistry, and liver function tests before treatment initiation. Ribociclib was initiated only in patients with a baseline corrected QT interval using Fridericia’s formula (QTcF) <450 ms.

Complete blood counts and liver function tests were performed every two weeks during the first two treatment cycles and prior to each subsequent cycle according to institutional practice. Electrocardiography was repeated on Day 14 of Cycle 1, at the beginning of Cycle 2, and thereafter when clinically indicated or in patients receiving concomitant medications associated with QT prolongation. Dose interruption, dose reduction, or permanent discontinuation was managed according to the approved prescribing information for ribociclib.

### Assessment

The primary endpoints were PFS, OS, and safety in the first-line metastatic setting. Tumor response was evaluated according to Response Evaluation Criteria in Solid Tumors (RECIST) version 1.1 where applicable, using radiologic imaging interpreted by the treating investigator in routine clinical practice. Imaging assessments were performed approximately every 3–4 months or earlier when disease progression was clinically suspected. For patients with bone-only or otherwise non-measurable disease, progression was determined based on imaging evidence. ER and PR expression were quantified by immunohistochemistry (IHC) and reported as the proportion of tumor cell nuclei exhibiting positive staining. ER and PR positivity were defined as nuclear staining in at least 1% of tumor cells. For inclusion in Cox regression models, ER and PR expression levels were coded as ordinal variables: 0 (negative, <1%, applied to PR), 1+ (low expression, 1–9%), 2+ (intermediate expression, 10–50%), and 3+ (high expression, >50%). PFS was defined as the interval from initiation of CDK4/6 inhibitor therapy to the date of radiologic or clinical disease progression, death from any cause, or the analysis cutoff date of December 1, 2024, whichever occurred first. OS was defined as the interval from CDK4/6 inhibitor initiation to the date of death from any cause or the analysis cutoff date, whichever occurred first. Disease-free interval (DFI) was defined as the time elapsed from completion of curative-intent treatment for early-stage breast cancer to the date of confirmed disease recurrence. Adverse events were graded throughout the treatment period according to the National Cancer Institute Common Terminology Criteria for Adverse Events, version 4.03 (NCI-CTCAE v4.03).

### Statistical analysis

All statistical analyses were performed using IBM SPSS Statistics, version 26. Continuous variables are reported as medians, and categorical variables as numbers and percentages. Survival outcomes were estimated using the Kaplan–Meier method and compared using the log-rank test. Cox proportional hazards regression was used to explore factors associated with PFS. Variables included in the multivariable Cox model were selected based on clinical relevance, availability in the dataset, and the results of univariable analysis. No automated stepwise selection procedure was used. Given the modest sample size, the multivariable analysis should be interpreted as exploratory. The proportional hazards assumption was checked graphically using log-minus-log survival plots. Missing data were not imputed; patients with missing values were excluded only from analyses involving those variables. Patients lost to follow-up were censored at the date of their last documented assessment. The numbers at risk at prespecified time points were derived from the Kaplan–Meier risk set. Exact PFS probabilities and corresponding 95% confidence intervals at 12 and 24 months were calculated from the Kaplan–Meier estimates. ER and PR expression levels were entered into the Cox model as ordinal variables, assuming a linear trend across categories. A two-tailed p-value <0.05 was considered statistically significant.

### Ethical approval

This retrospective study was approved by the Ethics Committee for Biomedical Research of Ho Chi Minh City Oncology Hospital, Vietnam (Approval No. 570A/BVUB-HĐĐĐ; December 12, 2022). Owing to the retrospective nature of the study and the use of anonymized medical records, the requirement for written informed consent was waived by the Ethics Committee in accordance with institutional policies and applicable regulations. Patient confidentiality was strictly maintained throughout the study, and all data were anonymized prior to analysis. The study was conducted in accordance with the ethical principles of the Declaration of Helsinki.

## Results

**Figure 1:**
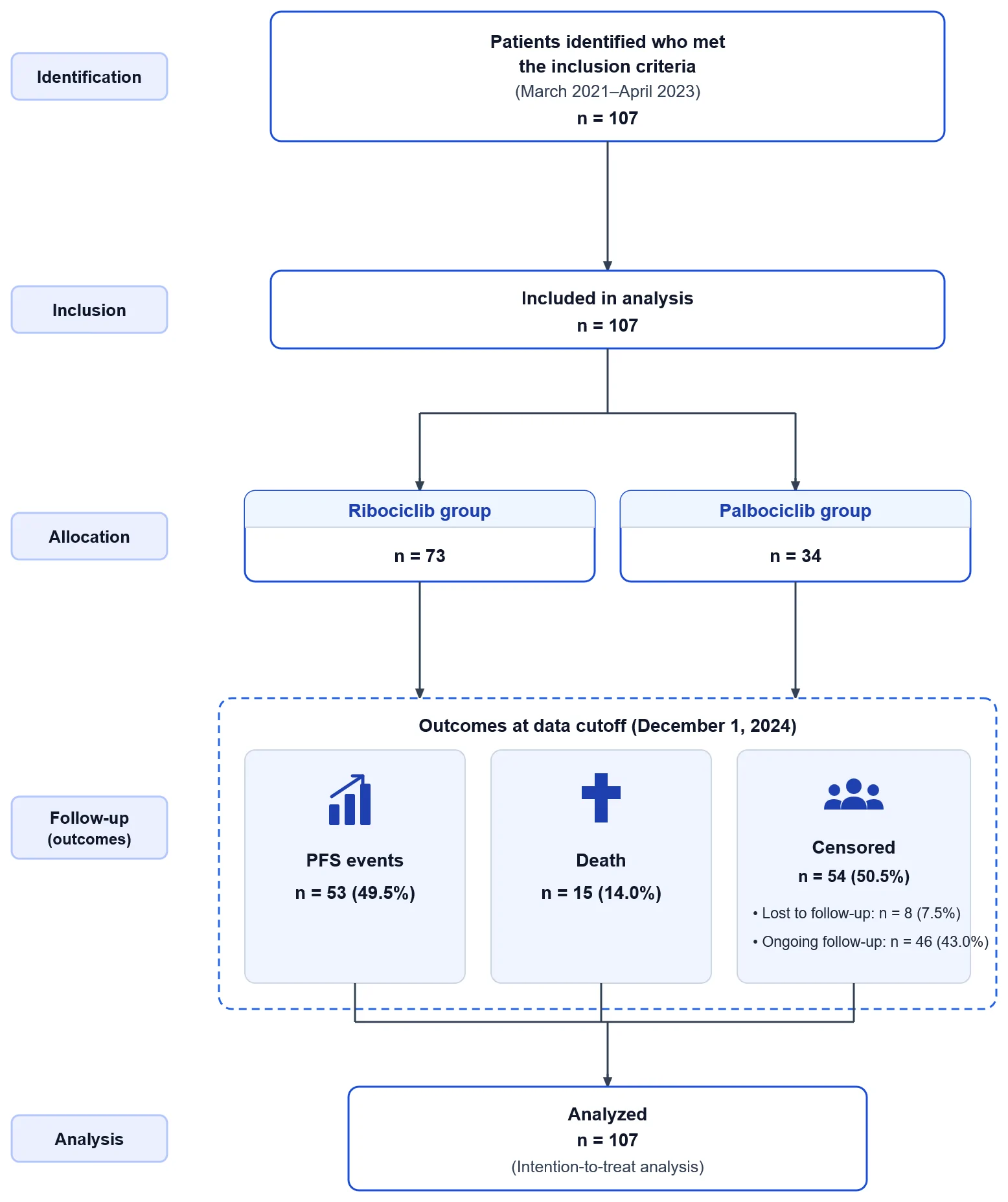
CONSORT-style patient flow diagram.

Between March 2021 and April 2023, a total of 107 patients participated in this study. The patient selection and analysis flow is shown in **Figure 1**. Patient characteristics are presented in **Table 1**. All patients were ER-positive, and 18.7% were PR-negative while retaining ER positivity.

**Table 1:** Baseline patient characteristics.

Characteristics	Total (N = 107)	Ribociclib (n=73)	Palbociclib (n=34)	p-value
Age group				0.85
<65 years	87 (81.3)	59 (80.8)	28 (82.4)	
≥65 years	20 (18.7)	14 (19.2)	6 (17.6)	
ECOG performance status				0.896
0	3 (2.8)	2 (2.7)	1 (2.9)	
1	92 (86.0)	62 (84.9)	30 (88.2)	
2	11 (10.3)	8 (11.0)	3 (8.8)	
3	1 (0.9)	1 (1.4)	0	
Histologic grade				0.313
Grade 1	6 (5.6)	5 (6.8)	1 (2.9)	
Grade 2	88 (82.2)	60 (82.2)	28 (82.4)	
Grade 3	10 (9.3)	5 (6.8)	5 (14.7)	
Unknown	3 (2.8)	3 (4.1)	0	
Ki67				0.761
<20%	40 (37.4)	28 (38.4)	12 (35.3)	
≥20%	67 (62.6)	45 (61.6)	22 (64.7)	
HER2 status				0.73
Negative	100 (93.5)	68 (93.2)	32 (94.1)	
HER2 1+	2 (1.9)	1 (1.4)	1 (2.9)	
HER2 2+/FISH−	5 (4.7)	4 (5.5)	1 (2.9)	
Menopausal status				0.851
Postmenopausal	55 (51.4)	36 (49.3)	19 (55.9)	
Premenopausal with bilateral oophorectomy	44 (41.1)	31 (42.5)	13 (38.2)	
Premenopausal receiving GnRH agonist	7 (6.5)	5 (6.8)	2 (5.9)	
Premenopausal after ovarian irradiation	1 (0.9)	1 (1.4)	0	
Disease setting				0.58
Recurrent metastatic disease	67 (62.6)	47 (64.4)	20 (58.8)	
*De novo* metastatic disease	40 (37.4)	26 (35.6)	14 (41.2)	
Disease-free interval				0.897
<12 months	5 (4.7)	4 (5.5)	1 (2.9)	
12–24 months	12 (11.2)	8 (11.0)	4 (11.8)	
>24 months	50 (46.7)	35 (47.9)	15 (44.1)	
*De novo* stage IV	40 (37.4)	26 (35.6)	14 (41.2)	
Number of metastatic sites				0.809
1	49 (45.8)	32 (43.8)	17 (50.0)	
2	36 (33.6)	25 (34.2)	11 (32.4)	
≥3	22 (20.6)	16 (21.9)	6 (17.6)	
Metastatic pattern				0.174
Bone-only metastasis	30 (28.0)	20 (27.4)	10 (29.4)	
Visceral metastasis	70 (65.4)	46 (63.0)	24 (70.6)	
Other	7 (6.5)	7 (9.6)	0	
Endocrine response status				0.678
Endocrine sensitive	66 (61.7)	46 (63.0)	20 (58.8)	
Endocrine resistant	41 (38.3)	27 (37.0)	14 (41.2)	
Endocrine therapy				0.372
Aromatase inhibitor	93 (86.9)	62 (84.9)	31 (91.2)	
Fulvestrant	14 (13.1)	11 (15.1)	3 (8.8)	

There were 7/107 cases (6.5%) with the first immunohistochemical results (in the early stage) belonging to the following groups: luminal B HER2-positive (1.9%), HER2-positive (0.9%), triple-negative (0.9%), and unknown immunohistochemistry (2.8%). All 7 cases had their lesions biopsied again when they relapsed for a second immunohistochemistry. The second immunohistochemistry results of the above group were: 2/107 cases (1.8%) were luminal A and 5/107 cases (4.7%) were luminal B HER2 negative. The median duration of CDK4/6 inhibitor treatment was 21.6 months. The majority of patients (86.9%) initiated treatment at the full recommended dose; initial dose reductions were attributable primarily to ECOG performance status 2 (4.7%) and advanced age (4.7%). The median time to first dose reduction was 11.6 weeks (range, 3–36 weeks), with the most common reasons being neutropenia (28.9%), elevated liver enzymes (2.8%), fatigue (1.9%), and anemia (1.9%). Dose interruptions were required in 63 patients (58.9%), with a median delay duration of 10.7 days (range, 7–28 days); the principal causes were neutropenia (53.2%), elevated liver enzymes (2.8%), anemia (1.9%), and pancytopenia (0.9%). Permanent treatment discontinuation occurred in 57 patients (53.3%), predominantly due to disease progression (49.5%), with 3.8% attributable to patient preference. Among patients who progressed on CDK4/6 inhibitor therapy, 45.3% continued to receive endocrine therapy thereafter.

With a data cutoff of December 1, 2024, the median follow-up for the entire cohort was 29 months. Among the 54 censored patients, the median follow-up duration was 29.5 months (range, 22–43 months). Eight of 107 patients (7.5%) were lost to follow-up and were censored at the date of their last documented assessment. At the data cutoff, 53 patients had experienced a PFS event and 54 patients were censored, including the eight patients lost to follow-up. The best overall response is summarized in **Table 2**. At the time of analysis, 15 patients (14.0%) had died. The median time from CDK4/6 inhibitor initiation to first objective response was 3.8 months. Treatment outcomes according to the CDK4/6 inhibitor are summarizedin **Table 3**.

**Table 2:** Best overall response.

Response	N (%)
Complete response	2 (1.9)
Partial response	28 (26.2)
Stable disease < 6 months	22 (20.6)
Stable disease ≥ 6 months	45 (42.1)
Disease progression	10 (9.3)
Overall response rate	30 (28.0)
Clinical benefit rate	75 (70.1)
Disease control rate	97 (90.7)

**Table 3:** Treatment outcomes according to CDK4/6 inhibitor.

Outcomes	Total	Ribociclib	Palbociclib	p-value
Median PFS, months	28.0	28.0	25.0	0.709
ORR	30 (28.0)	22 (30.1)	8 (23.5)	0.479
CBR	75 (70.1)	52 (71.2)	23 (67.6)	0.706
Any adverse event	107 (100.0)	73 (100.0)	34 (100.0)	N/A
Grade 3–4 adverse event	69 (64.5)	50 (68.5)	19 (55.9)	0.204
Neutropenia	99 (92.5)	68 (93.2)	31 (91.2)	0.804
Grade 3–4 neutropenia	63 (58.8)	45 (61.6)	18 (52.9)	—
Anemia	38 (35.5)	25 (34.2)	13 (38.2)	0.877
Thrombocytopenia	24 (22.4)	15 (20.5)	9 (26.5)	0.312
AST elevation	21 (19.6)	16 (21.9)	5 (14.7)	0.479
ALT elevation	17 (15.9)	13 (17.8)	4 (11.8)	0.579
Dose interruption	63 (58.9)	46 (63.0)	17 (50.0)	0.203
Dose reduction	38 (35.5)	30 (41.1)	8 (23.5)	0.139
Permanent discontinuation	57 (53.3)	38 (52.1)	19 (55.9)	0.712

Permanent discontinuation was mainly due to disease progression; no patient discontinued permanently because of toxicity.

**Figure 2:**
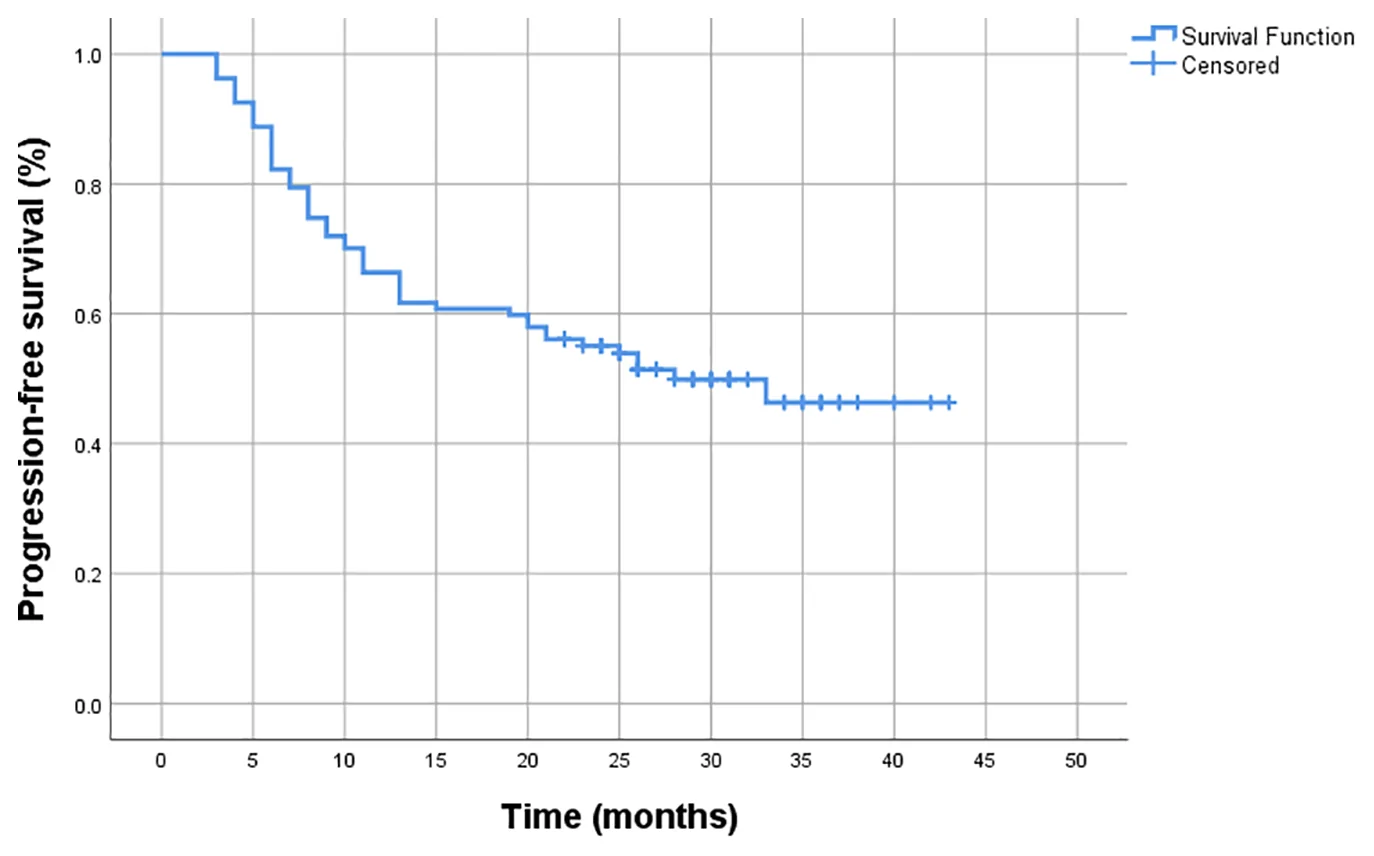
Kaplan-Meier curves of PFS.

The median duration of response was 25 months. The overall response rate (ORR) was 28.0%, and the clinical benefit rate (CBR) was 70.1%. The median PFS for the overall cohort was 28.0 months. The Kaplan–Meier-estimated PFS rates were 66.4% (95% CI, 56.6%–74.4%) at 12 months and 54.1% (95% CI, 44.2%–63.0%) at 24 months. The Kaplan–Meier curve for PFS is shown in **Figure 2**. The corresponding numbers at risk were 71 and 51 patients, respectively. Multivariable analysis identified ER and PR expression levels as independent prognostic factors for PFS. The results of the univariable and multivariable Cox regression analyses are presented in **Table 4**. In subgroup analyses, patients with endocrine-sensitive disease experienced significantly longer PFS than those with endocrine-resistant disease (median PFS, not reached vs. 13.0 months; log-rank p < 0.001).

**Table 4:** Multivariate Cox regression analysis of prognostic factors for PFS.

Characteristic	Comparison grouping	Univariate HR (95% CI)	p	Multivariate HR (95% CI)	p
Estrogen-receptor status	1+/2+/3+	0.277 (0.129–0.595)	**0.001**	0.268 (0.108–0.661)	**0.004**
Progesterone-receptor status	0/1+/2+/3+	0.669 (0.535–0.837)	**<0.001**	0.703 (0.537–0.919)	**0.010**
Metastatic site	Bone metastasis only/visceral metastasis/other locations	1.107 (0.698–1.756)	0.665	0.837 (0.500–1.400)	0.497
DFI	<12 months/12–24 months/>24 months/*de novo* stage IV	0.791 (0.576–1.087)	0.148	1.209 (0.664–2.203)	0.535
Age group	<65/≥65	0.733 (0.345–1.557)	0.420	0.809 (0.351–1.863)	0.619
HER2 status	Negative/1+/2+ and FISH negative	0.472 (0.158–1.407)	0.178	0.431 (0.145–1.280)	0.130
Histological grade	1/2/3/unknown	1.690 (1.093–2.612)	**0.018**	1.608 (0.990–2.613)	0.055
Ki67	<20/≥20	0.822 (0.476–1.420)	0.483	0.692 (0.386–1.241)	0.217
Disease setting	IV/recurrence	1.743 (0.958–3.171)	0.069	1.427 (0.535–3.806)	0.477
CDK4/6 inhibitors	Ribociclib/palbociclib	1.102 (0.619–1.964)	0.741	1.739 (0.922–3.280)	0.087
Endocrine therapy	Aromatase inhibitor/fulvestrant	1.300 (0.610–2.769)	0.497	1.452 (0.610–3.458)	0.400
CDK4/6 inhibitor dose reduction	No reduction/1 dose reduction/2 dose reductions	1.554 (0.955–2.528)	0.076	1.479 (0.900–2.429)	0.122

ER and PR expression levels were modeled as ordinal variables. The multivariable Cox model was exploratory because of the modest sample size.

Bold values indicate statistical significance (p < 0.05).

Patients without visceral metastases and those with *de novo* metastatic disease demonstrated a trend toward longer PFS compared with patients with visceral metastases and recurrent metastatic disease, respectively; however, these differences did not reach statistical significance. Median PFS was not reached versus 20.0 months for patients without versus with visceral metastases (p = 0.082), and not reached versus 21.0 months for patients with *de novo* versus recurrent metastatic disease (p = 0.069).

No significant difference in PFS was observed between patients with bone-only disease and those with non-bone-only disease (median PFS, not reached vs. 23.0 months; log-rank p = 0.252).

**Figure 3:**
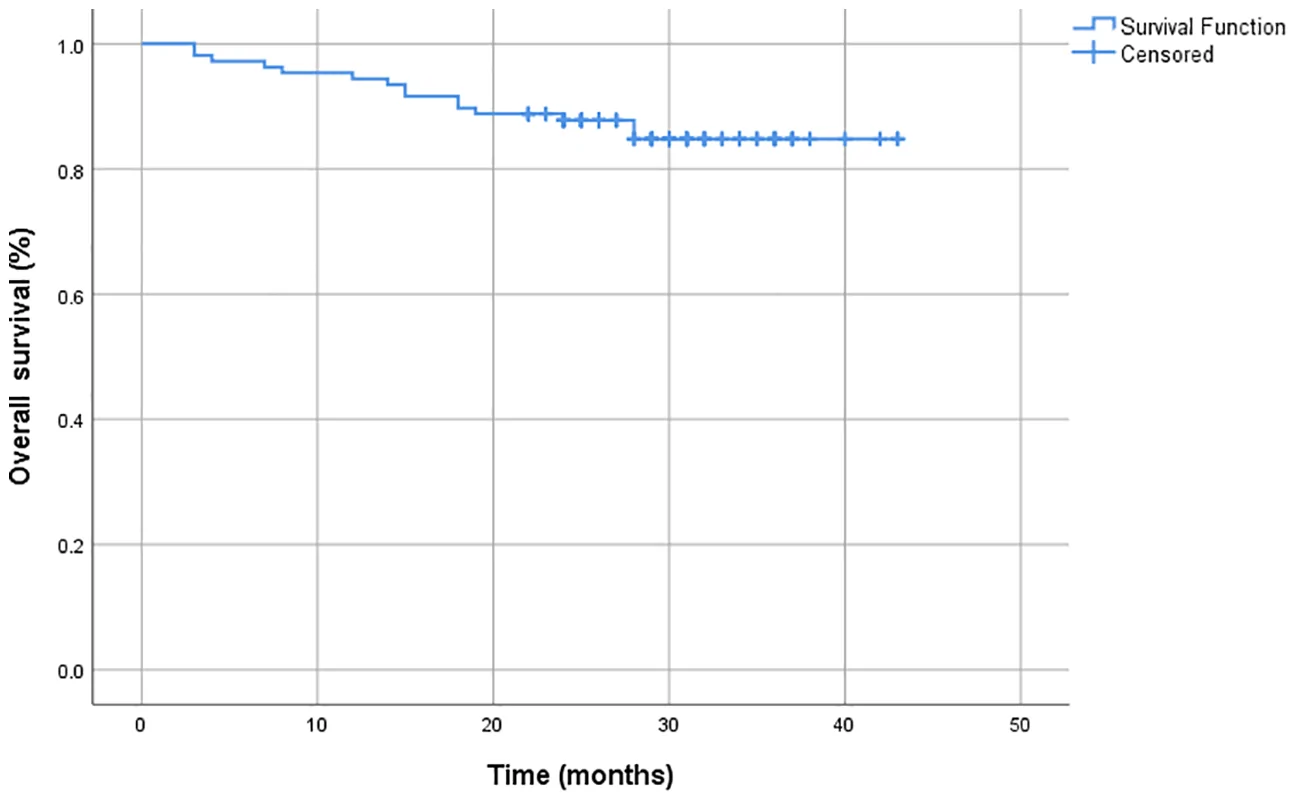
Kaplan-Meier curves of OS.

As of December 1, 2024, a total of 15 patients (14.0%) had died. The median OS was not reached; the 12- and 24-month OS rates were 88.8% and 87.8%, respectively. The Kaplan–Meier curve for OS is shown in **Figure 3**. Because only 14% of patients in the study died, we did not analyze the association of OS with associated factors due to the low mortality rate. The median time to first adverse event was 4 weeks. Toxicity per patient is calculated as the highest peak toxicity across treatment cycles. There were 58.8% of cases of grade 3, 4 neutropenia, and no cases had fever. Grade 3 thrombocytopenia and hemoglobin reduction were 3.7% and 1.9%, respectively, with no grade 4 toxicity. Non-hematologic toxicities were predominantly mild; grade 3 fatigue was documented in 2.8% of patients, and grade 3 AST/ALT elevation in 3.7%. No patient in the ribociclib cohort developed a QTcF interval exceeding 480 milliseconds.

## Discussion

### Patient characteristics

The median age of patients in our cohort was 52 years, with only 18.7% aged ≥65 years. This finding is consistent with the Vietnamese real-world study reported by Le Thanh Duc et al. (9), but differs substantially from the PALOMA-2 and CompLEEment-1 studies, in which 40.8% and 33.1% of patients, respectively, were aged ≥65 years (5, 10). These findings suggest that patients with HR-positive/HER2-negative metastatic breast cancer treated in Vietnam are generally younger than those reported in Western populations.

Approximately half of the study population was naturally postmenopausal, whereas the remaining premenopausal patients received ovarian function suppression through bilateral oophorectomy, ovarian irradiation, or gonadotropin-releasing hormone agonists. The proportion of patients requiring ovarian function suppression was considerably higher than that reported by Low et al. (24%) (11) and Porte et al. (18.4%) (12), likely reflecting the younger age distribution of our cohort.

Our study results recorded 67/107 cases (62.6%) of breast cancer relapse after adjuvant treatment, and only 37.4% of cases were stage IV from the beginning. This result is consistent with findings from other international studies. The PALOMA-2 study recorded 68.7% of cases as patients who relapsed after adjuvant treatment (13). The MONALEESA-2 study reported recurrence rates of 65.9% and stage IV rates of 34.1% from the beginning.

This study recorded 15.9% of cases with DFI ≤ 24 months. The MONALEESA-2 study surveyed 668 patients, with 5.4% of cases having DFI ≤ 24 months. In the CompLEEment-1 study, the DFI ≤ 24 months was 11.8% (10). This difference may partly reflect earlier relapse patterns in the Vietnamese population or referral selection bias.

Compared with PALOMA-2 and CompLEEment-1 (5, 10), our cohort had a notably lower rate of involvement of three or more organ sites, while the proportion with bone-only metastasis was comparable (**Supplementary Table 1****).**

Several published real-world studies have reported PR-negative rates ranging from 17.7% (CompLEEment-1 (10)) to 21.7% (Fountzilas et al. (14)). The PR-negative rate in our cohort was 18.7%, with all PR-negative cases retaining ER positivity. Regarding HER2 expression, 93.5% of patients had HER2 IHC 0 disease, while 6.5% (7/107) had HER2-low disease, including 1.9% with HER2 1+ and 4.7% with HER2 2+/FISH-negative tumors. This low HER2-low prevalence aligns with the Singaporean real-world experience reported by Low et al. (4%) (11). In contrast, recent Western real-world studies have reported substantially higher HER2-low prevalence. For example, Lapuchesky et al. reported a HER2-low rate of 34.4% (HER2 1+: 23.1%; HER2 2+: 11.3%) (15). Zattarin et al. similarly reported HER2-low rates as high as 62.8% (16). In the P-VERIFY real-world study evaluating three CDK4/6 inhibitors, the median age was 66 years; 9% of patients were male, 63% were White, 50.8% had stage IV disease at diagnosis, 74.6% were postmenopausal, and 19.1% were premenopausal. Median follow-up was 33.0 months for palbociclib plus an aromatase inhibitor, 15.7 months for ribociclib plus an aromatase inhibitor, and 21.5 months for abemaciclib plus an aromatase inhibitor (17). The endocrine resistance rate in our cohort (38.3%) was broadly consistent with those reported internationally, including 32% in Lee et al. (18) and 29.7% in Porte et al. (12).

### Treatment outcomes

Ribociclib was the predominant CDK4/6 inhibitor in our cohort (68.2%), with palbociclib accounting for the remaining 31.8%, a distribution concordant with institutional data reported by Le Thanh Duc et al. (9). However, in Jia Li Low’s study (11), 95% used palbociclib, while in Erik’s study (19), 93.6% were treated with palbociclib. The reason for this difference may be because the FDA approved palbociclib in 2015, while ribociclib was approved in 2017 and abemaciclib in 2018. The two studies by foreign authors, which started in 2015, may have led to a higher rate of palbociclib use compared to other CDK4/6 inhibitors.

Our study found that only 86.9% of cases received full doses of CDK4/6 inhibitors from the beginning, which may be attributed to the fact that the performance status of patients in our study was higher than in similar studies conducted in France and China. 

The median follow-up time of this study was 29 months, which is quite similar to the study by Raffaella (20), which had a median follow-up time of 24 months, and in the MONALEESA-2 study, it was 26.4 months (6).

The median time from the start of CDK4/6 inhibitors to the first response was 3.8 months. This result is consistent with many other studies worldwide. According to author Sonia Pernas (21), the median time was 3.7 months. The RIGHT Choice study recorded a median time to response of 4.7 months (22).

The ORR was 28.0% in the overall population and 31.5% among patients with measurable disease, both of which were lower than the corresponding rates in MONALEESA-2 (40.7% and 52.7%, respectively) (23). Differences in patient selection between randomized trials and routine clinical practice may also have contributed. This difference may be attributable to disparities in DFI: MONALEESA-2 enrolled a higher proportion of patients with a DFI exceeding 24 months (60.5%) compared with our cohort (46.7%), and longer DFI is generally associated with greater endocrine sensitivity and a higher likelihood of objective response.

The Kaplan–Meier–estimated median PFS of 28 months in our cohort compares favorably with those reported in pivotal randomized trials despite the inclusion of a substantial proportion of patients with recurrent disease. (PALOMA-2: 24.8 months (5); MONALEESA-2: 25.3 months (6)) and in real-world registries (RIBANNA: 31.7 months (24)) (**Supplementary Table 2****).**

The median OS was not reached in our study, reflecting the relatively short follow-up and low number of death events at the current analysis date. The 12- and 24-month OS rates of 88.8% and 87.8%, respectively, are comparable to estimates from other studies. Real-world data from the UK, by G. Gullick, on 666 patients showed that the median OS in the palbociclib group was 47 months (25). The MONALEESA-2 study had a median OS of 63.9 months (26). In the P-VERIFY study, no statistically significant difference in OS was observed among the CDK4/6 inhibitors, and the proportions of patients alive at 12, 24, and 30 months were similar across treatment groups (**Supplementary Table 3**). The effectiveness of CDK4/6 inhibitor-based therapy observed in our cohort is also supported by other real-world studies across different populations and healthcare settings (27–30).

### Safety

The safety profile observed in our study was consistent with the established toxicity profile of CDK4/6 inhibitors. Grade 3–4 neutropenia was the most common adverse event but was not associated with any cases of febrile neutropenia. Severe nonhematologic toxicities were uncommon, and the incidence of grade 3–4 hematologic and nonhematologic adverse events was generally comparable with previously published real-world studies (**Supplementary Tables 4**, **5**). No patient experienced febrile neutropenia or clinically significant QTc prolongation, supporting the feasibility of routine toxicity monitoring in clinical practice. These findings support the favorable tolerability of CDK4/6 inhibitor therapy in routine clinical practice.

### Strengths and limitations

This study has several strengths. It represents one of the largest real-world cohorts evaluating first-line CDK4/6 inhibitor therapy for HR-positive/HER2-negative metastatic breast cancer in Vietnam. In addition, all patients received CDK4/6 inhibitors as first-line systemic therapy for advanced disease, and endocrine resistance was defined according to internationally accepted ABC5 criteria, thereby improving the consistency and clinical relevance of the analyses.

Nevertheless, several limitations should be acknowledged. First, the retrospective single-center design is inherently subject to selection bias, information bias, and unmeasured confounding. Tumor response and disease progression were assessed in routine clinical practice by treating physicians in conjunction with attending radiologists, rather than through a central independent radiologic review. Consequently, inter-observer variability and potential assessment bias may have affected the evaluation of ORR, CBR, and PFS, particularly in patients with bone-only or non-measurable disease. Therefore, these efficacy outcomes should be interpreted with appropriate caution. Second, treatment allocation to ribociclib or palbociclib was not randomized but was influenced by physician preference, drug availability, reimbursement policies, patient affordability, and access to patient assistance programs, introducing the possibility of treatment-selection bias. Third, the relatively small sample size limited the statistical power for subgroup analyses and comparisons between treatment groups. Fourth, median overall survival was not reached because only 15 deaths had occurred at the time of data cutoff, precluding robust analyses of prognostic factors for OS. Finally, the findings may not be fully generalizable to other institutions because this was a single-center study conducted in a tertiary oncology hospital in Vietnam.

Despite these limitations, our study provides valuable real-world evidence regarding the effectiveness and safety of first-line CDK4/6 inhibitor therapy in a Southeast Asian population and complements evidence derived from randomized clinical trials.

## Conclusion

In this real-world cohort of 107 patients with HR-positive, HER2-negative recurrent or metastatic breast cancer treated with first-line ribociclib or palbociclib combined with aromatase inhibitor or fulvestrant at Ho Chi Minh City Oncology Hospital, the median PFS was 28 months over a median follow-up of 29 months. The Kaplan–Meier-estimated PFS rates at 12 and 24 months were 66.4% (95% CI, 56.6%–74.4%) and 54.1% (95% CI, 44.2%–63.0%), respectively. The 12- and 24-month OS rates were 88.8% and 87.8%, respectively; the median OS was not reached. Endocrine-resistant disease was associated with significantly shorter PFS in the Kaplan–Meier subgroup analysis, whereas differences according to visceral metastasis, disease presentation, and bone-only disease were not statistically significant. On multivariate Cox regression analysis, ER and PR expression levels were independently associated with PFS outcomes. The combination of CDK4/6 inhibitors and endocrine therapy had a manageable safety profile, with no cases of febrile neutropenia. No new toxicities related to CDK4/6 inhibitors other than those already known emerged.

## Data Availability

The raw data supporting the conclusions of this article will be made available by the authors, without undue reservation.

## Supplementary material

Supplementary Table 1 Recurrence characteristics compared with other studies.

Supplementary Table 2 Comparison of initial CDK4/6 inhibitor doses with other authors.

Supplementary Table 3 OS compared with other authors.

Supplementary Table 4 Grade 3, 4 hematological toxicities compared with other authors.

Supplementary Table 5 Grade 3–4 non-hematological toxicities compared with other studies.
