# Supplementary material for: First-line CDK4/6 inhibitor treatment of HR-positive, HER2-negative metastatic breast cancer: real-world data from Ho Chi Minh City Oncology Hospital, Vietnam
Source: Front Oncol. 2026 Sep 7;16:1871703. doi: 10.3389/fonc.2026.1871703 (PMC13598703; doi:10.3389/fonc.2026.1871703)
Supplement: Supplementary file 1 [file Table1.docx]

**SUPPLEMENTARY MATERIAL**

**First-Line CDK4/6 Inhibitor Treatment of HR-Positive, HER2-Negative Metastatic Breast Cancer: Real-World Data from Ho Chi Minh City Oncology Hospital, Vietnam**

**Supplementary Table S1. Recurrence characteristics compared with other studies**

| **Characteristic** | **PALOMA-2 [5] (N=666), %** | **CompLEEment-1 [9] (N=3,246), %** | **Our study (N=107), %** |
| --- | --- | --- | --- |
| **Number of metastatic sites** |  |  |  |
| 0 | — | 0.5 | 0 |
| 1 | 31.1 | 27.8 | 45.8 |
| 2 | 26.4 | 28.4 | 33.6 |
| ≥3 | 42.5 | 43.3 | 20.6 |
| **Metastatic site** |  |  |  |
| Bone-only metastasis | 23.2 | 21.7 | 28.0 |
| Visceral metastasis | 48.2 | 61.4 | 65.4 |
| Other/non-visceral | 28.6 | 16.9 | 6.5 |

**Supplementary Table S2. Comparison of initial CDK4/6 inhibitor doses with other studies**

| **Initial dose** | **Porte et al. [10] (N=310), %** | **Zhong et al. [19] (N=64), %** | **Our study (N=107), %** |
| --- | --- | --- | --- |
| Full recommended starting dose | 95.2 | 92.2 | 86.9 |
| One-level reduced starting dose | 4.5 | 7.8 | 10.3 |
| Two-level reduced starting dose | 0.3 | — | 2.8 |

**Supplementary Table S3. Overall survival compared with other studies**

| **Study** | **12-month OS, %** | **24-month OS, %** |
| --- | --- | --- |
| Waller et al. [25] (N=162) | 89.0 | — |
| Rugo et al. [26] (N=2,888) | — | 76.6 |
| Our study (N=107) | 88.8 | 87.8 |

**Supplementary Table S4. Grade 3–4 hematological toxicities compared with other studies**

| **Toxicity** | **Fountzilas et al. [14] (N=365), %** | **Our study (N=107), %** |
| --- | --- | --- |
| Leukopenia | 21.1 | 14.0 |
| Neutropenia | NR | 58.8 |
| Febrile neutropenia | NR | 0 |
| Hemoglobin decrease/anemia | 1.9 | 1.9 |
| Thrombocytopenia | 1.9 | 3.7 |

**S**

**upplementary Table S5. Grade 3–4 non-hematological toxicities compared with other studies**

| **Toxicity** | **Fountzilas et al. [14] (N=365), %** | **Our study (N=107), %** |
| --- | --- | --- |
| Stomatitis | 0.3 | 0 |
| Skin rash | 1.4 | 0 |
| Diarrhea | 1.1 | 0 |
| AST/ALT elevation | NR | 3.7 |
| Clinically significant QTc prolongation | NR | 0 |

**Abbreviations:** ALT, alanine aminotransferase; AST, aspartate aminotransferase; NR, not reported; OS, overall survival; QTcF, corrected QT interval using Fridericia’s formula.
